# Serum markers in early-stage and locally advanced melanoma

**DOI:** 10.1007/s13277-015-3564-2

**Published:** 2015-05-23

**Authors:** Iwona Lugowska, Maria Kowalska, Małgorzata Fuksiewicz, Beata Kotowicz, Ewa Mierzejewska, Hanna Koseła-Paterczyk, Katarzyna Szamotulska, Piotr Rutkowski

**Affiliations:** 1 Department of Soft Tissue/Bone Sarcoma and Melanoma, Maria Sklodowska Curie Memorial Cancer Centre and Institute of Oncology, K.W. Roentgen Street 5, Warsaw, Poland; 2 Department of Epidemiology, Institute of Mother and Child, M. Kasprzak Street 17a, Warsaw, Poland; 3 Department of Pathology and Laboratory Diagnostics, Laboratory of Tumor Markers, Maria Sklodowska Curie Memorial Cancer Centre and Institute of Oncology, K.W. Roentgen Street 5, Warsaw, Poland

**Keywords:** Melanoma, Prognosis, Early stage, Serum

## Abstract

The identification of prognostic factors in cutaneous melanoma allows choosing the most effective treatment, especially in group of patients with locoregional disease. Markers related to carcinogenesis and angiogenesis in particular have effect on the course of the disease. The aim of this study was to evaluate clinical utility of vascular endothelial growth factor (VEGF), matrix metalloproteinase 2 (MMP-2), MMP-9, tissue inhibitors of metalloproteinase 1 (TIMP-1), and YKL-40 in serum of melanoma patients at pathological stages I–III. We included 148 adult patients with melanoma. The median follow-up was 40 months. Disease recurrence was observed in 43 patients; 3-year disease-free survival (DFS) rate was 71.7 %; 35 patients died; and the 3-year overall survival (OS) rate was 85 %. Concentrations of VEGF, MMP-2, MMP-9, TIMP-1, and YKL-40 were measured by ELISA kits. VEGF, MMP-9, TIMP-1, and YKL-40 were significantly higher in group of patients than in controls. Increased concentrations of TIMP-1 were related to patient survival, which in the group of lower and increased TIMP-1, disease-free survival amounted to 81 vs. 61 % (*p* = 0.014) and overall survival −88 vs. 82 % (*p* = 0.050), respectively. An increased concentration of YKL-40 was observed in 59 % of patients with ulceration and in 26 % of patients without ulceration (*p* = 0.012). We have found a clinically significant correlation between YKL-40 and MMP-9 (rho = 0.363; *p* = 0.004) as well as YKL-40 and VEGF (rho = 0.306; *p* = 0.018). In melanoma patients at stages I–III, the high concentrations of TIMP-1 in serum predicted adverse prognosis. YKL-40 was associated with ulceration of primary tumor, which is a very important prognostic factor.

## Introduction

Melanoma is a malignant tumor deriving from neuroectodermal melanocytes, and it is one of the most aggressive cancers in humans. Depending on the clinical stage determined in accordance with the classification of the American Joint Committee on Cancer (AJCC), patients are qualified to prognostic groups and linked to the optimal scheme of treatment. The AJCC classification is based on the following clinicopathological factors: depth of invasion (Breslow’s depth), ulceration, mitotic index, lymph nodes involvement, presence of distant metastases, as well as concentration of lactate dehydrogenase (LDH) [1]. Although the AJCC classification correctly stratifies patients to the risk groups, there is still 50 % of patients with locoregional stage who recur. Therefore, it is worth to consider to add other prognostic factors which are, e.g., related to tumor biology.

Neoangiogenesis is one of the most important processes of tumor development, and vascular endothelial growth factor (VEGF) is a key factor stimulating endothelial cell proliferation, survival, and migration, as well as leading to the increase in permeability of blood vessels. The process of neoangiogenesis is modulated by the activity of matrix metalloproteinases (MMPs) including MMP-2 and MMP-9, and their tissue inhibitor of metalloproteinase 1 (TIMP-1) [2]. The glycoprotein YKL-40, known as chitinase 3-like 1 (CHI3L1), also increases the migration of endothelial cells and leads to VEGF over-expression [3].

The effect of MMP activity is the degradation of extracellular matrix and plasma membrane of cells which activates the release of VEGF, bFGF, and other factors related to neoangiogenesis. MMPs (MMP-2 (gelatinase A) and MMP-9 (gelatinase B)) degrade type IV collagen—the main component of extracellular matrix and the basement membrane of vessels. This process enables dissemination of tumor cells to circulation. TIMPs including TIMP-1 also play a significant role in tumor development through participation in the degradation of extracellular matrix, in the process of neoangiogenesis and apoptosis, as well as in proliferation of normal and tumor cells [4, 5].

In neoangiogenesis, YKL-40—a 38-kDa glycoprotein binding heparin (gp38k) is released by the number of cells such as chondrocytes, smooth muscle cells, active neutrophils, macrophages, tumor-associated macrophages (TAM), and tumor cells, and is characterized by multidirectional activity such as involvement in the neoangiogenesis and reconstruction of extracellular matrix [2].

So far, published data has demonstrated that the tissue over-expression of VEGF, MMP-2, MMP-9, and TIMP-1 has an adverse effect on the course of the disease and survival of melanoma patients [6–9]. Only several publications have concerned the evaluation of the utility of measurements of VEGF, MMP-2, MMP-9, TIMP-1, and YKL-40 in serum in melanoma; what is more, the results are controversial [10–17].

The aim of the study was to define clinical utility of serum biomarkers: VEGF, MMP-2, MMP-9, TIMP-1, and YKL-40 in patients with skin melanoma at locoregional stage.

## Patients and methods

### Study population

This prospective study included melanoma patients treated in the Department of Soft Tissue/Bone Sarcoma and Melanoma in Maria Sklodowska Curie Memorial Cancer Center and Institute of Oncology (COI), in Warsaw, Poland between 2009 and 2010. We included 148 adult patients (median age 55 years) with pathologically confirmed primary melanoma at stages I–III. Patients were treated according to the national guidelines: sentinel lymph node biopsy (SLNB) with excision of the primary tumor (or scar after excisional biopsy) was performed [18]. Patient characteristics are presented in Table 1. Median follow-up was 40 months. The study has been approved by the local bioethics committee.

**Table 1:** Clinical characteristics of the study population

Patient characteristic (*n* = 148)	Number of patients (%)
Gender	
Male	74 (50.0)
Female	74 (50.0)
Age (years)	
Median (range)	55 (20–76)
Anatomic site of primary tumor	
Trunk	61 (41.2)
Lower extremity	51 (34.5)
Upper extremity	23 (15.5)
Other	13 (8.8)
Primary tumor status	
pT1	33 (22.3)
pT2	44 (29.7)
pT3	42 (28.4)
pT4	29 (19.6)
Ulceration	
Present	69 (46.6)
Absent	75 (50.7)
Unknown	4 (2.7)
Lymph node status	
pN0	103 (69.6)
pN1	27 (18.2)
pN2	9 (6.1)
pN3	9 (6.1)
Clinical stage	
I	55 (37.2)
II	48 (32.4)
III	45 (30.4)
Follow-up (months)	
Median (range)	40 (2–58)

### Healthy controls

The ranges of serum markers were determined in 50 subjects (median age 42 years, range 19–74 years). They were all healthy (not taking any medicine, without any clinical signs or symptoms of cancer, liver, joints, metabolic or hormonal disease).

## Methods

Venous blood was taken from patients before surgical treatment. In order to standardize clotting conditions, all sera were separated within 1 h after blood collection and stored in −70 °C until assayed. Concentrations of VEGF, MMP-2, MMP-9, and TIMP-1 were measured by ELISA kits of R&D Systems, Minneapolis, USA. YKL-40 was determined by ELISA assays (Quidel, Santa Clara, CA, USA). Normal levels of serum VEGF, MMP-2, MMP-9, TIMP-1, and YKL-40 had been previously determined in 50 healthy volunteers. The number of patients analyzed with each biomarker was different due to the limited amount of serum stored from each patient in our tissue bank. The numbers of examined samples were as follows in patient group: VEGF—148, TIMP-1—146, MMP-2—113, MMP-9—113, and YKL-40—60, and in the control group: VEGF—50, TIMP-1—48, MMP-2—40, MMP-9—38, and YKL-40—18.

### Statistical analysis

Patient demographics, tumor characteristics, and treatment details were analyzed in a descriptive manner. To analyze the differences of variables between the groups, the chi-square test for categorical variables, and the Mann-Whitney *U* test, the Kruskal-Wallis test, or Dunn post hoc test for continuous variables were applied. The correlation coefficients between serum markers were calculated using the Spearman’s rank correlation, and rho above 0.3 was considered as clinically significant. In the analyses of prognostic role of serum markers in survival, the cut-off point for YKL-40 equal 80 ng/ml was based on published data [19], whereas for VEGF, MMP-9, and TIMP-1, the medians of these parameters from our melanoma patients were used as cut-off points. The disease-free survival (DFS) time was calculated from the start of SLNB/lymphadenectomy to the most recent follow-up or disease recurrence, while the overall survival (OS) time from SLNB to the most recent follow-up or death. The impact of clinicopathological and biochemical factors on DFS and OS was estimated in the univariate analyses according to Kaplan-Meier method, and log-rank tests were used for comparisons, while in the multivariate analyses, Cox proportional hazards regression model was applied.

Statistical calculations were performed using IBM-SPSS v.18.0 software. The differences were considered as statistically significant if *p* value was <0.05.

## Results

### The serum biomarkers in melanoma and control group

The patients with melanoma in comparison with the reference group had significantly higher median concentrations of VEGF (399 vs. 94 pg/ml; *p* = 000.1), MMP-9 (741 vs. 415 ng/ml; *p* < 0.001), TIMP-1 (161 vs. 116 ng/ml; *p* < 0.001), and YKL-40 (59 vs. 43 ng/ml; *p* = 0.001). The concentrations of MMP-2 were similar in these both groups (increased values only in three patients). Therefore, MMP-2 was excluded from further statistical analyzes.

**Fig 1:**
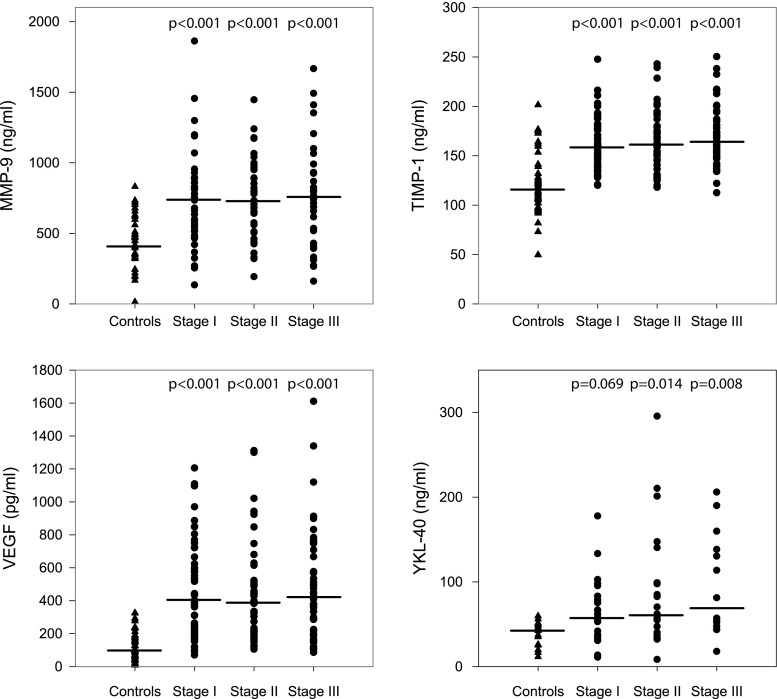
The MMP-9, TIMP-1, VEGF, and YKL-40 serum level distributions in the melanoma patients at stages I, II, and III and in the control group. The *horizontal lines* indicate medians in particular groups; all *p* values concern the comparisons with the control group

In pairwise comparisons of the patients with melanoma at stages I, II, and III with control group, all differences in VEGF, MMP-9, TIMP-1, and YKL-40 serum levels between patients and controls were statistically significant except for the difference in YKL-40 concentration between melanoma stage I patients and healthy individuals (Fig. 1). In melanoma patients, median levels of the studied biomarkers did not differ between consecutive clinical stages I–III.

The increased value of YKL-40 was observed in 59 % of patients with ulceration, whereas only in 26 % of patients without ulceration (*p* = 0.012). No significant correlations appeared between concentrations of TIMP-1, MMP-9, and VEGF and clinicopathological parameters.

### The correlations between serum markers in melanoma

**Fig. 2:**
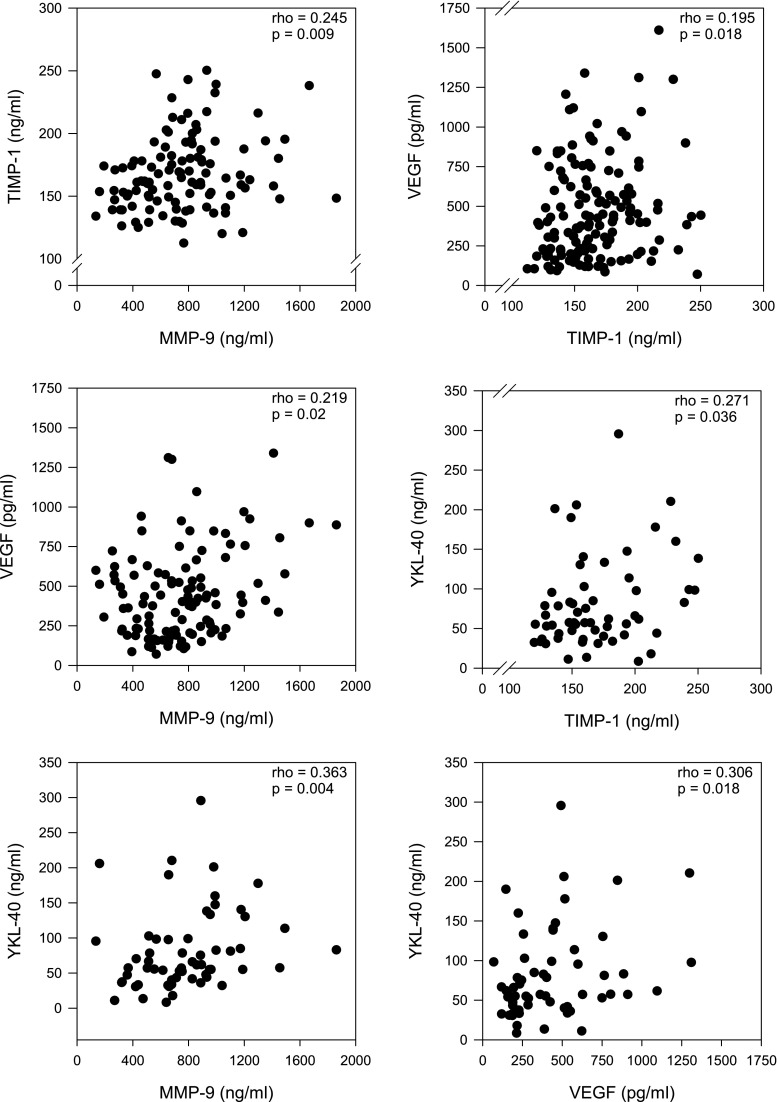
Correlations between MMP-9, TIMP-1, VEGF, and YKL-40 serum levels in melanoma patients

While analyzing the relationship between concentrations of biomarkers, the most significant values of correlation coefficient were determined between YKL-40 and MMP-9 (rho = 0.363; *p* = 0.004) as well as YKL-40 and VEGF (rho = 0.306; *p* = 0.018). Remaining correlation coefficients assumed to be not clinically important ranged from 0.195 to 0.271 (Fig. 2).

### Clinical prognostic factors in melanoma

Disease recurrence after primary treatment was diagnosed in 43 patients (29.1 %); 3-year DFS rate was 71.7 % and 5-year DFS rate was 60 %. Thirty five patients died at the time of analysis; the 3-year OS rate was 85 %, while 5-year OS rate was 58.5 %.

**Fig. 3:**
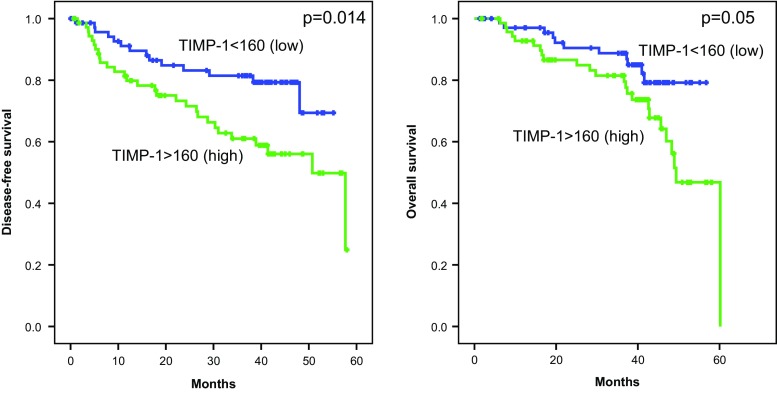
Three-year disease-free survival and 3-year overall survival in melanoma patients depending on TIMP-1 level

In those patients in which recurrence appeared, the concentrations of the inhibitor of metalloproteinase TIMP-1 were significantly higher than in the group without recurrence (*p* = 0.045); the similar observation was done in patients who died (*p* = 0.034) in comparison to the patients who survived. Kaplan-Meier analysis of survival demonstrated that in the group of patients with increased concentration of TIMP-1 at the time of diagnosis, the percentage of survivors was significantly lower than in group where the concentration of TIMP-1 was below cut-off point. This observation concerned both 3-year DFS and 3-year OS, which were 61 vs. 81 % (*p* = 0.014) and 82 vs. 88 % (*p* = 0050), respectively (Table 2, Fig. 3).

**Table 2:** Clinicopathological and biochemical factors influencing 3-year disease***-***free survival and 3-year overall survival of melanoma patients

Patient characteristics	3-year DFS (%)	*p* value	3-year OS (%)	*p* value
Gender				
Female	76.3	0.040	86.6	0.103
Male	67.0		83.9	
Age at the time of the diagnosis				
≤55 years	75.7	0.310	87.1	0.664
>55 years	67.0		83.2	
Anatomic site of primary tumor				
Trunk	74.8	0.992	92.2	0.789
Upper extremity	72.7		79.7	
Lower extremity	69.9		84.8	
Other	64.8		64.8	
Ulceration				
Yes	84.1	0.003	94.9	0.002
No	60.8		76.4	
Breslow scale				
pT1	89.8	<0.001	96.6	0.012
pT2	72.4		89.3	
pT3	71.1		83.5	
pT4	52.1		70.4	
Lymph node status				
pN0	80.8	<0.001	93.2	<0.001
pN1	66.2		82.9	
pN2	44.4		77.8	
pN3	22.2		29	
Clinical stage				
I	87.2	<0.001	97.8	<0.001
II	74.0		88.3	
III	51.4		68.1	
TIMP-1				
≤160 ng/ml (low)	81.4	0.014	88.8	0.050
>160 ng/ml (high)	61.0		81.5	
YKL-40				
≤80 ng/ml (low)	77.0	0.164	92.5	0.111
>80 ng/ml (high)	59.4		78.9	
VEGF				
≤399 pg/ml (low)	72.0	0.799	82.3	0.616
>399 pg/ml (high)	71.5		88.2	
MMP-9				
≤741 ng/ml (low)	68.8	0.583	80.8	0.258
>741 ng/ml (high)	71.5		89.7

**Fig. 4:**
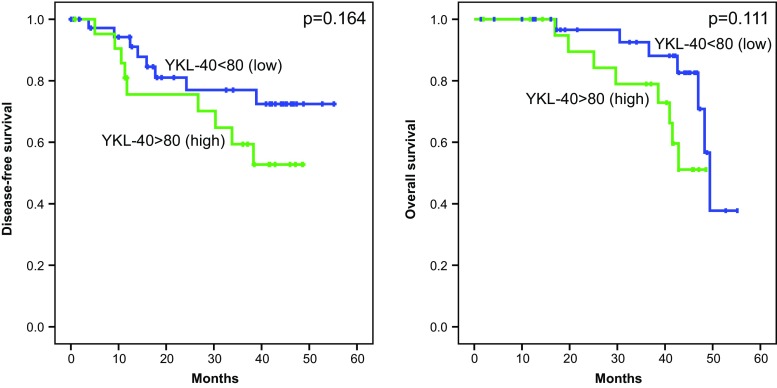
Three-year disease-free survival and 3-year overall survival in melanoma patients depending on YKL-40 level

A relation between the concentration of YKL-40 and disease recurrence has also been observed. In the group with higher YKL-40 level, the recurrence was observed in 53 % of patients, while in the group with lower YKL-40, the recurrence was observed only in 30 % of them, although *p* value did not reach statistical significance (*p* = 0.090). In case of increased concentration of YKL-40, the survival rate was lower than in group with YKL-40 below 80 ng/ml. Observed differences in the 3-year DFS and 3-year OS rates amounted to 59.4 vs. 77 % and 78.9 vs. 92.5 %, respectively. The difference was not statistically significant, probably due to the small sample size (Table 2, Fig. 4).

A univariate analysis confirmed prognostic role of well-known clinical factors such as depth of infiltration, nodal involvement, ulceration (parameters included in the AJCC classification), and TIMP-1 (Table 2). In multivariate Cox regression model, factors significantly associated with 3-year DFS remain pN (HR = 1.87, *p* = 0.007) and TIMP-1 level >160 ng/ml (HR = 2.03, *p* = 0.041), while factor significantly associated with 3-year OS was only pN (HR = 2.3, *p* = 0.002).

## Discussion

The effect of the interaction between cancer cells and tumor microenvironment very often results in increased concentrations of numerous biomarkers in serum. Biomarkers which concentration is related to clinicopathological parameters or survival may act as tumor markers. In the presented study, we have evaluated a clinical utility of the concentrations of VEGF, MMP-2, MMP-9, TIMP-1, and YKL-40 in serum of patients with skin melanoma at locoregional disease. The concentrations of VEGF, MMP-9, TIMP-1, and YKL-40 were significantly higher in patients with melanoma than in control group. From analyzed biomarkers, increased concentrations of TIMP-1 appeared to be a negative prognostic factor for both DFS and OS. It has been also demonstrated that increased concentrations of YKL-40 are caused by the presence of ulceration.

In melanoma, various studies dedicated to VEGF concentration in serum and its clinical utility were reviewed by Dewing D et al. [20]. Authors concluded that elevated serum levels of VEGF strongly correlated with disease progression and poor prognosis. Biological background of this observation is linked to the modulation of angiogenic switch and changes appearing in cytokine expression levels during tumor transition from radial to invasive vertical and then metastatic growth. The interest in VEGF is related to its priority role in the process of neoangiogenesis, as well as with available target therapies blocking its activity. In compliance with literature data and results of presented study, the concentrations of VEGF in sera of melanoma patients were significantly higher than in control groups [10, 21–23].

Although clinical utility of VEGF in serum was the subject of many publications, the results were ambiguous. Tas et al. [23] noticed that VEGF concentration increased along with the depth of the invasion. In our study, we have not detected any dependencies between the concentrations of VEGF and the depth of invasion. The lack of particular relationship between the concentrations of VEGF and the depth of invasion, as well as gender, age, location of primary site, lymph nodes involvement, and clinical stages has been demonstrated by Pelletier et al. [10] and Tas et al. [22].

The prognostic role of VEGF concentration in melanoma was pointed by Ascierto et al. [11]. Authors showed that the increased VEGF was related to the progression-free survival, but not to patients’ overall survival. In our opinion, the prognostic role of VEGF in serum is still unknown and further studies are required.

The transitional research demonstrated that VEGF stimulates endothelial cells to release MMPs. MMPs degrade extracellular matrix and plasma membrane, as well as they enable to release growth factors responsible for migration of endothelial and cancer cells. The key role is assigned to MMP-2 and MMP-9. The tissue expression of those MMPs has an effect on clinical data, for example, the increase of MMP-2 has been observed in patients at higher clinical stages, whereas the expression of MMP-9 is combined with lower clinical stages [4, 8, 20]. Only few publications concerned the evaluation of the concentrations of MMP-2 and MMP-9 in serum of patients with melanoma. In our study, the concentrations of MMP-2 did not differentiate patients from control group. However, the concentrations of MMP-9 were significantly higher in those with melanoma. Nevertheless, we did not demonstrate an association between the concentration of MMP-9 and clinicopathological parameters, as well as DFS and OS. Contrary to our study, Nikkola et al. [12] presented data according to which MMP-9 had an effect on survival, although these data included only patients with advanced/metastatic melanoma.

Since the relation between the increase in the activity of MMPs and the progression of melanoma seems to be quite obvious, the role of tissue inhibitors of metalloproteinases (TIMPs) is not easy to explain. The activity of TIMPs is related not only to the regulation of the reconstruction of extracellular matrix but also to the regulation of cellular processes. Observed dependencies between over-expression of TIMPs and increase in tumor burden have confirmed the hypothesis of TIMP role in tumor progression [24].

We have confirmed that the concentration of TIMP-1 was significantly higher in melanoma patients than in control group and that this increase has an effect on DFS and OS in melanoma at locoregional stage. These data were compliant with results of other researches, also devoted to patients with metastatic disease [25]. However, in contrary to Yoshino et al. [25], we have not observed any associations between the concentration of TIMP-1 and the depth of invasion, clinical stage, or nodal status. Those findings are consistent with data presented by Tas et al. [14]. It is also worth mentioning that we have not confirmed the relation between TIMP-1, age, and ulceration, which was done by Tas et al. [14]. The utility of prognostic role of TIMP-1 is a subject of research since, along with other parameters, it may be a useful tool for monitoring treatment and predicting the course of the disease [15, 26].

The effect of TIMPs on the disease progression is probably related to their mechanisms of action regulated by the activation of surface receptors. Therefore, TIMPs may act directly on proliferation, migration, or angiogenesis, as well as indirectly through regulation of MMP activity. Using those mechanisms, TIMP-1 may mutually promote growth, as well as may restrain the proliferation of tumor cells and regulate apoptosis and angiogenesis [5].

Another biomarker involved in neoangiogenesis, inflammation, and reconstruction of extracellular matrix is glycoprotein YKL-40. In our group of patients, the concentration of YKL-40 was higher in those who recurred, as well as in those in which the observation ended with death (the differences were not statistically significant probably due to sample size). In the paper presented by Schmidt et al. [16] which included 234 patients with stage I (*n* = 162) and II (*n* = 72), serum YKL-40, along with thickness and ulceration, was an independent prognostic factor of relapse-free survival and overall survival. However, the concentrations of YKL-40 were not related to the presence of ulceration, which is not consistent with results obtained by us. Egberts et al. [27] showed that in melanoma patients, serum levels of YKL-40, S-100B, and LDH correlated significantly with the stage of disease. In stage IV melanoma, only S100-B significantly (not YKL-40) correlates with treatment response and survival. Because the data of prognostic role of YKL-40 in melanoma are disputable, further studies are needed [17].

The analysis of the interaction between serum biomarkers revealed the most significant correlation coefficient between YKL-40, VEGF, and MMP-9. The participation of YKL-40 in the processes of neoangiogenesis is the effect of researches performed over the past few years. Based on paper presented by Shao et al. [3], YKL-40 may not only play a role in neoangiogenesis acting as a potent angiogenic factor capable of stimulating tumor vascularization but also may induce FAK-MAPK by signaling and up-regulating VEGF receptor 2 in endothelial cells. YKL-40 stimulates endothelial cells to migration showing similar activity as VEGF [28].

The studies on the YKL-40 mechanisms of action, as well as its utility in anti-YKL-40 therapies have been the matter of recent studies [29]. Perhaps the results will allow to explain not only the mode of action of YKL-40 which remains enigmatic but also the discrepancy between published papers. Riabov et al. [29] assumed that the most important are overlapping mechanisms such as biology of tumor cells, reaction of immune system, as well as dependence between YKL-40 and TAM. Salamon J et al. [30] on the other side presented very interesting results with antibody against YKL-40. The aim of their study was to evaluate the effect of anti-YKL-40 monoclonal antibody on tumor growth and morphology in xenograft model of human melanoma, as well as pancreatic adenocarcinoma in scid mice. Authors found that monotherapy with antibody targeting YKL-40, rapidly enhances melanoma tumor size by increasing the formation of new tumor vessels. They concluded that YKL-40 could still be an interesting target for anti-tumor therapy, but first, the mode of action of YKL-40 should be solved.

## Summary

The aim of presented study was to evaluate the clinical utility of serum concentrations of VEGF, MMP-2, MMP-9, TIMP-1, and YKL-40 in patients with melanoma at locoregional stage. Marker selection was based on their role in cancerogenesis and the possibility of the application of targeted therapy [30–32]. We have proved that increased concentrations of TIMP-1 were unfavorable prognostic factors for both disease-free survival and overall survival, as well as for relation between YKL-40 and ulceration. We have also observed a trend related to YKL-40 and recurrence. In order to verify obtained results, further researches are required.
